# The Biosafety Research Road Map: The Search for Evidence to Support Practices in the Laboratory—Zoonotic Avian Influenza and *Mycobacterium tuberculosis*

**DOI:** 10.1089/apb.2022.0038

**Published:** 2023-09-12

**Authors:** Stuart D. Blacksell, Sandhya Dhawan, Marina Kusumoto, Kim Khanh Le, Kathrin Summermatter, Joseph O'Keefe, Joseph Kozlovac, Salama Suhail Almuhairi, Indrawati Sendow, Christina M. Scheel, Anthony Ahumibe, Zibusiso M. Masuku, Allan M. Bennett, Kazunobu Kojima, David R. Harper, Keith Hamilton

**Affiliations:** ^1^ Mahidol-Oxford Tropical Research Medicine Unit, Faculty of Tropical Medicine, Mahidol University, Bangkok, Thailand.; ^2^ Centre for Tropical Medicine and Global Health, Nuffield Department of Medicine, Nuffield Department of Medicine Research Building, University of Oxford, Oxford, United Kingdom.; ^3^ Institute for Infectious Diseases, University of Bern, Bern, Switzerland.; ^4^ Ministry for Primary Industries, Wellington, New Zealand.; ^5^ United States Department of Agriculture, Agricultural Research Service, Beltsville, Maryland, USA.; ^6^ Abu Dhabi Agriculture and Food Safety Authority, Abu Dhabi, United Arab Emirates.; ^7^ Research Center for Veterinary Science, National Research and Innovation Agency, Indonesia.; ^8^ WHO Collaborating Center for Biosafety and Biosecurity, Office of the Associate Director for Laboratory Science, Center for Global Health, Centers for Disease Control and Prevention, Atlanta, Georgia, USA.; ^9^ Nigeria Centre for Disease Control and Prevention, Abuja, Nigeria.; ^10^ National Institute for Communicable Diseases of the National Health Laboratory Services, Johannesburg, South Africa.; ^11^ UK Health Security Agency, London, United Kingdom.; ^12^ Department of Epidemic and Pandemic Preparedness and Prevention, World Health Organization (WHO), Geneva, Switzerland.; ^13^ The Royal Institute of International Affairs, London, United Kingdom.; ^14^ World Organisation for Animal Health (OIE), Paris, France.

**Keywords:** Zoonotic avian influenza, *Mycobacterium tuberculosis*, pathogen characteristics, biosafety evidence, biosafety knowledge gap

## Abstract

**Introduction:**

The Biosafety Research Road Map reviewed the scientific literature on a viral respiratory pathogen, avian influenza virus, and a bacterial respiratory pathogen, *Mycobacterium tuberculosis.* This project aims at identifying gaps in the data required to conduct evidence-based biorisk assessments, as described in Blacksell et al. One significant gap is the need for definitive data on *M. tuberculosis* sample aerosolization to guide the selection of engineering controls for diagnostic procedures.

**Methods:**

The literature search focused on five areas: routes of inoculation/modes of transmission, infectious dose, laboratory-acquired infections, containment releases, and disinfection and decontamination methods.

**Results:**

The available data regarding biosafety knowledge gaps and existing evidence have been collated and presented in Tables 1 and 2. The guidance sources on the appropriate use of biosafety cabinets for specific procedures with *M. tuberculosis* require clarification. Detecting vulnerabilities in the biorisk assessment for respiratory pathogens is essential to improve and develop laboratory biosafety in local and national systems.

## Introduction

The World Organisation for Animal Health (WOAH), The World Health Organization (WHO), and Chatham House have collaborated to improve the sustainable implementation of laboratory biological risk management, particularly in low-resource settings. The Biosafety Research Roadmap (BRM) project aims at supporting the application of laboratory biological risk management and improving laboratory sustainability by providing an evidence base for biosafety measures (including engineering controls) and evidence-based biosafety options for low-resource settings.

This will inform strategic decisions on global health security and investments in laboratory systems. This work involves assessing the current evidence base required for implementing laboratory biological risk management, aiming at providing better access to evidence, identifying research and capability gaps that need to be addressed, and providing recommendations on how an evidence-based approach can support biosafety in low-resource settings.

## Materials and Methods

A 15-member technical working group (TWG) was formed to develop a BRM to support the application of laboratory biological risk management and improve laboratory sustainability by providing an evidence base for biosafety measures. The TWG conducted a gap analysis for a selected list of priority pathogens on procedures related to diagnostic testing and associated research for those pathogens, including but not limited to sample processing, testing, animal models, tissue processing, necropsy, culture, storage, waste disposal, and decontamination.

The TWG screened databases, websites, publications, reviews, articles, and reference libraries for relevant data to achieve this. The main research domains used to perform the literature searches were the ABSA International database, Belgian Biosafety Server, Centers for Disease Control and Prevention (CDC) reports, WHO reports, PubMed, and internet searches for terms related to biosafety matters, including, for example, inactivation, decontamination, laboratory-acquired infections, laboratory releases, and modes of transmission.

The summary of evidence and potential gaps in biosafety was divided into five main sections: route of inoculation/modes of transmission, infectious dose, laboratory-acquired infections, containment releases, and disinfection and decontamination strategies. Blacksell et al.^1^ describe the materials and methods and explain why the gap analysis was performed. Here, we present the general characteristics of Zoonotic avian influenza and *Mycobacterium tuberculosis*, the current biosafety evidence, and available laboratory-acquired infections and laboratory releases.

### Zoonotic Avian Influenza Virus

#### General characteristics

Zoonotic avian influenza (ZAI) viruses are Type A Influenza viruses and members of the *Orthomyxoviridae* family, with segmented genomes of eight negative-sense, single-stranded RNA. ZAI viruses are considered Risk Group3 pathogens in most jurisdictions.^2^ Variations in antigenicity and phylogenetics of the haemagglutinin (HA) and neuraminidase (NA) proteins allow them to be divided into subtypes designated H(x)N(y) with 18HA and 11 NA subtypes have been identified.^3^

Variations in the virus occur by antigenic drift and antigenic shift. Antigenic drift occurs when mutations result in changes to the HA and NA proteins and is a continuous process as the virus replicates in the host. Antigenic shift occurs only in influenza A viruses, resulting in a complete exchange of HA and NA genes proteins, creating novel virus strains.^4^ When these novel viruses can infect humans, they can cause pandemics because the population has limited immunity.

Antigenic shift can occur when an unknown animal strain jumps to humans or can result from a reassortment of the segmented genome of two or more influenza viruses within the same cell. Compared with antigenic drift, which is a continual process, antigenic shift occurs more infrequently and has caused four pandemics over the past 100 years (1918, 1957, 1968, and 2009).^5^

Virus strains are designated highly pathogenic avian influenza (HPAI) when, or if, the HA cleavage site is polybasic and is recognized by ubiquitous cellular proteases, leading to systemic and often fatal infection in poultry.^3^ Low-pathogenic avian influenza (LPAI) causes localized and limited infection and has an HA cleavage site identified only by trypsin-like proteases.^3^ Only LPAI subtypes H5 and H7 have displayed this phenotype in natural isolates.^3^

Designation as HPAI or LPAI is also determined by in vivo studies in poultry or *in ovo* in embryonated chicken eggs using the intravenous pathogenicity index (IVPI). The HPAI reservoir is largely wild (aquatic) birds such as ducks and geese,^6^ although the possibility exists for transspecies infection to other mammals depending on subtype and strain.^6^ Outbreaks of HPAI H5N1 were reported in Hong Kong in 1997, and since 2003, this virus has spread globally (from Asia to Europe to Africa and North America), becoming endemic in poultry in some regions.^6^

Given next are two examples regarding the severity of outbreaks and resultant culling in farmed poultry. During 2022, HPAI outbreaks in the US have approached record numbers of birds affected compared with previous outbreaks, with over 49 million birds in 46 states either dying due to infection or being culled following exposure to infected birds.^7^ Similarly, Europe has reported a surge in HPAI cases in poultry between September 2021 through October 2022, affecting 37 countries and resulting in the culling of 50 million birds.

Continued circulation of HPAI is ongoing through December 2022 and is attributed to the spread from waterfowl.^8^ In addition, a variant in a mink farm outbreak may have public health implications; although the initial source was also attributed to waterfowl, mink-to-mink transmission occurred, indicating that a single mutation may have resulted in mammal-to-mammal transmission.^8^

#### Treatment and prophylaxis

Annual seasonal influenza vaccination is available for those potentially exposed to ZAI virus in occupational or community settings.^9–11^ However, seasonal vaccines are developed annually to protect against strains projected to dominate human seasonal influenza, not ZAI virus, so the seasonal influenza vaccination available may or may not be effective against a given ZAI virus strain. Several anti-viral treatments are available for influenza infections, such as oseltamivir, zanamivir, M2 inhibitors, amantadine, and rimantadine.^12^

#### Diagnostics

In the laboratory, several methods are used to diagnose and characterize the ZAI virus. Molecular techniques such as reverse transcriptase polymerase chain reaction are used to identify and type the virus, followed by culture in embryonated chicken eggs^13–15^ to determine pathogenicity by IVPI. Serological tests can determine H and N types; hemagglutination inhibition and neuraminidase inhibition, respectively. Enzyme-linked immunosorbent assays are widely used, although they cannot generally discriminate between virus groupings (influenza A, influenza B, etc.). IVPI tests are commonly used for animal infection studies^6^ or to determine whether control methods are necessary.

### Biosafety Evidence

#### Mode of transmission

The mode of transmission for the ZAI virus is mainly from bird to human,^16,17^ although fomites may also play a role.^18^ The disease is transmitted via aerosols^19^ and large droplets.^19,20^ Transmission of the ZAI virus to humans is usually from direct contact with infected animals or contaminated environments; however, human-to-human transmission of avian influenza has been observed.^15^

#### Infectious dose

The minimum infectious dose for ZAI viruses in humans is not well characterized. The 50% human infectious dose (HID_50_) for influenza was mainly determined using attenuated vaccine strains and may not be representative of disease transmission. Such virus strains may not accurately represent the HID_50_ of wild-type strains that may be more virulent than the attenuated vaccine strain.^21^ A review of minimum HID_50_ of human influenza viruses demonstrated a range from 1 × 10^3^ to 1 × 10^5^ 50% tissue culture infectious dose (TCID_50_) depending on the strain and the subtype.^21^ The use of carefully titrated viral stocks determined that the HID_50_ was 0.6–3.0 TCID_50_ via the inhalation route,^19^ and using intranasal drops, the HID_50_ was determined to be 127–320 TCID_50_.^19^

#### Laboratory-acquired infections

There are no published reports of laboratory-acquired ZAI virus infections. While not strictly laboratory-acquired, it was reported that a veterinarian was infected with the influenza A (H7N2) virus from cats in a shelter in New York, USA, resulting in zoonotic transmission.^22^

#### Disinfection and decontamination

There are numerous methods for the effective inactivation of the ZAI virus, and this has been covered in the comprehensive review of the subject by De Benedictis et al.^23^

##### Chemical

There are numerous effective chemical disinfection methods for ZAI viruses. The choice of chemical and methodology is often dependent on the circumstances of the disinfection requirement.^23^ In the review of De Benedictis et al.,^23^ effective chemicals for ZAI virus disinfection include acids (2–5% hydrochloric acid, 50 ppm hypochlorous acid, 0.2% citric acid, 1250–5000 ppm potassium monopersulfate, and 2% cresolic acid), alkalis (0.1 mol/L NaOH), chlorine (2–3% calcium hypochlorite and sodium hypochlorite), peroxide (3–6% hydrogen peroxide and 2%Virkon^®^), aldehydes (1–2% glutaraldehyde, and 40% formalin), and ethanol (70% ethanol)-based disinfectants as well as soaps and detergents. However, the contact times and applications differ^23^ (Table 1).

**Table 1.** Detailed pathogen biosafety evidence for Zoonotic avian influenza

***Overview of the evidence and potential gaps in biosafety***				
***Method***	***Details***	***Evidence (direct quote where available)***	***Reference***	***Evidence gap? (yes/no)***
Modes of transmission	Bird to human	“The sequence comparison of HK/156 and CK/HK/220 H5N1 influenza viruses showed without doubt that the viruses had a closely related common ancestor, and the chicken virus serves as a reasonable progenitor virus to study the species jump for the human influenza virus.”	^17^	No
		“The observed H5 seroprevalence rate of 10% among PWs is high, compared with the other Hong Kong cohorts studied during 1997 by use of similar antibody testing methods. Seroprevalence rates among groups not exposed to infected human case patients and with presumed low levels of poultry exposure were 0% among adult blood donors and 0.7% among health care workers.”	^16^	
	Contact with fomites	“These observations suggest that the transmission of virus from donors who are shedding large amounts could occur for 2–8 hr via stainless steel surfaces and for a few minutes via paper tissues. Thus, under conditions of heavy environmental contamination, the transmission of influenza virus via fomites may be possible.”	^18^	No
	Aerosol	“Many, possibly most, natural influenza infections occur by the aerosol route and that the lower respiratory tract may be the preferred site of initiation of the infection.”	^19^	No
Infectious dose	Aerosol inoculation 50% HID_50_ = 0.6–3.0 TCID_50_	“The use of carefully titrated viral stocks enabled the determination of the minimal infectious dose by aerosol inoculation. For volunteers who lacked detectable neutralizing antibodies at the onset, the 50% human infectious dose (HID_50_) was 0.6–3.0 TCID_50_, if one assumes a retention of 60% of the inhaled particles.”	^19^	No
	Large droplets	“The HID_50_ measured when inoculation was performed by intranasal drops was 127–320 TCID_50._”	^19^	
	Wild-type virus	HID_50_ data have been generated using attenuated virus, which may not accurately reflect infectious dose of wild-type virus.		Yes
LAIs	None reported thus far	There are reports of veterinarians infected with Avian Influenza A (H7N2) Virus following exposure to sick cats. There are no reports of laboratory acquired ZAI.	^22^	Yes
Chemical inactivation	Hypochlorous acid solution 50 ppm	“In the present study, the aqueous phase of the original solution containing a free available chlorine concentration of 50 ppm could reduce the titer of an ordinary AIV (H7N1) from 10^7.7^ TCID_50_/ml to lower than the detectable limit within 5 sec, which is faster than in previous reports, and its harvested solution after spraying from a distance of 1 cm had the same ability, but it lost its efficacy after spraying from a distance of 30 cm.”	^25^	No
	Hydrochloric acid 2–5%—10 min Citric acid 0.2%—30 min	“Among the vast group of acid disinfectants, only two molecules (hydrochloric and citric acid) are widely used due to their mild corrosive effect and also due to higher safety for staff compared with the other acids. However, no specific experimental data are available on the efficacy of these compounds against AIVs.”	^23^	
	Calcium hypochlorite 2–3% Sodium hypochlorite—10–30 min	“This group is also efficacious against AIVs, but they require a thorough cleaning of all the surfaces on which they are going to be used. For this kind of compounds (i.e., sodium hypochlorite), addition of methyl glycol with anti-freeze action does not decrease the disinfectant activity, allowing for the use of these molecules also during winter. When these types of disinfectant are handled, their limitations must be taken into account in order to ensure optimal efficacy and also their safety for staff. In fact, they are corrosive for metals, pH dependent and inhibited by the presence of organic material.”	^23,97,98^	
	Hydrogen peroxide 3–6%—30 min Virkon^®^ 2%	“The activity of oxidizing compounds also decreases in the presence of organic residues. They cause irritation to mucus membranes, eyes and skin. Among this group, Virkon (Antec International Ltd., Suffolk, UK) is one of the most efficacious products against AIVs. However, reports on the specific efficacy against AIVs of hydrogen peroxide are contradictory, and for this reason additional information on its viricidal efficacy is necessary.”	^23^	
		“The resistance of a Pakistan isolate H7 subtype to Virkon-S activity was tested at different concentrations and incubation times. Four HA units of virus were put in contact with Virkon-S solution in peptone water at pH 7.0 at the disinfectant final dilutions of 0.5% (w/v), 1% and 2% and incubated for 30, 60, 90 and 120 min. All concentrations were able to inactivate AIV. Briefly, a concentration of 0.5% of Virkon-S solution was able to inactivate AIV fully after 90 min, while at 1% and 2% concentration complete inactivation was achieved after just 30 min.”	^23,28^	
	Glutaraldehyde 1–2%—30 min	“Aldehydes are widely used disinfectants for fumigation procedures. This group comprises two well-known compounds, formaldehyde and glutaraldehyde, both efficacious against AIVs. The efficacy of formaldehyde with the addition of a QAC has been tested successfully on AIVs. Glutaraldehyde is preferred to formaldehyde because it has a lower toxicity and the shortest residual activity, making it easy to handle product. However, its use may be limited by the high cost.”	^23,97^	
		“The disinfectant effect of glutaraldehyde is achieved at a dilution ranging from 1% to 2%, following an exposure time of 10–30 min. It is chemically stable and mildly corrosive for metals. In recent times, it has been widely used for chemical sterilization of medical instruments. However, for large-scale decontamination, it is not recommended due to its cost.”	^99^	
	Formalin 0.04–0.1%—16 h	“Formalin at low concentrations, such as 0.04% and 0.1%, is able to inactivate both HPAI and LPAI viruses (H5N2 A/chicken/Pennsylvania/1370/83, H5N9 A/turkey/Wisconsin/68 and H9N2 A/turkey/Wisconsin/68) after 16 h at 37°C, but preserves their hemagglutinating activity.”	^100^	
	Formalin 0.06–0.24%—12 h	The efficacy of formalin has also been tested at other dilutions against the H7N3 subtype (strain not reported). Three different dilutions (0.06%, 0.12%, and 0.24%) were tested at different incubation times (6, 12, 18 and 24 h). After 6 h, formalin was not able to inactivate AIV at a concentration of 0.06% and 0.12% and at a concentration of 0.24% no virus was detected by virus isolation. A time span of 12 h was necessary to inactivate AIV at all the tested concentrations.	^28^	
	Ethanol 70%—5–15 min	“Alcohols are efficacious against AIVs and other enveloped viruses. Their efficacy decreases suddenly at concentrations below 50% (v/v). Alcohols are inflammable and damage plastic objects and therefore they are not recommended for wide disinfection of poultry premises but only for restricted use on staff and laboratory decontamination. In fact, alcohols are actually employed as a thinner of other chemical disinfectants and several formulations containing this kind of compound are available for skin and laboratory equipment.”	^23^	
	Ethanol 70%—15 min	“Ethanol at a concentration of 70% (v/v) can inactivate four different AIV strains H7N2 subtype (A/chicken/PA/3972–1/97 with a titre of 10^5.5^ELD_50_/ml; A/chicken/PA/3972–2/97 with a titre of 10^4.5^ELD_50_/ml; A/chicken/PA/3779–1/97 with a titre of 10^4.5^ ELD_50_/ml; A/chicken/PA/3779–2/97 with a titre of 10^.4.5^ELD_50_/ml) after 15 min of contact time.”	^23,101^	
	55% w/w ethanol, 10% w/w propan-1-ol, 5.9% w/w propan-1.2-diol and 5.7% w/w butan-1.3-diol with 0.7% phosphoric acid	“A new disinfectant for personal use containing four alcohols (55% w/w ethanol, 10% w/w propan-1-ol, 5.9% w/w propan-1.2-diol and 5.7% w/w butan-1.3-diol) with 0.7% phosphoric acid has recently been tested for viricidal activity against several viruses. Its efficacy has been tested against a human type A influenza strain (Aichi/2/68 H3N2) at an initial titre of 10^7.5^CCID_50_/ml (dose infecting 50% of cell cultures). The results of three different assays demonstrated that the product at a dilution of 96% and 20% was able to inactivate the virus after 30 s and 1 min, respectively, both in the presence or on the absence of loaded protein (0.2% of bovine serum albumin or 10% of foetal calf serum).”	^102^	
	Cresolic acid 2%—10 min	“Another class of disinfectants largely used and efficacious against AIVs is the phenol compound group. The compounds belonging to this group have the following qualities: disinfectant effect in the presence of organic materials and low cost. The toxicity of these compounds changes from a high degree for O-phenyl phenol to a low degree for phenol crystal, the latter being most widely used among the phenol products. The disinfectant activity of these compounds in inactivating AIVs was tested and their efficacy at the recommended dilution also in combination with different anti-freeze molecules was demonstrated.”	^23,28,103^	
	PMPS Organic materials 5000 ppm—5 s 2500 ppm—5 s 1250 ppm—30 s 625 ppm—15 min No organic material 625 ppm—10 min Infected Rayon sheet 5000 ppm—30 s 2500 ppm—5 min 1250 ppm—15 min	“Regarding AIV, PMPS at 5,000, 2,500 and 1,250 ppm could inactivate AIV even in the presence of organic materials within 5 sec, 5 sec and 30 sec, respectively. At 625 ppm, PMPS could inactivate AIV within 10 min in the absence of organic material; however, in the presence of organic material, it could not inactivate AIV within 15 min.” “5,000, 2,500 and 1,250 ppm of PMPS could inactivate AIV… These results suggest that PMPS can be applied as a disinfectant or a virucidal agent that can inactivate AIV in contaminated carpets, clothes, towels, or bedding, especially in animal farms or hospitals.”	^24^	
	Soap, detergent and alkali—5 min	Soap (lifebuoy^®^), detergent (surf excel^®^), and alkali (caustic soda) destroyed infectivity after 5 min at 0.1, 0.2, and 0.3% dilution.	^27^	
Thermal inactivation	63°C, 2 min	“In good accordance with these reports, the HPAI A virus (H7N7) examined in our study was inactivated at 63°C and ambient pressure in 2 min.”	^32^	No
	56°C, 30 min 28°C, 1 day	“H5N1 virus lost infectivity after 30 min at 56°C, after 1 day at 28°C but remained viable for more than 100 days at 4°C.”	^27^	
	70°C, 5 min 80°C, 2.5 min 90°C, 1 min	“H1N1 was inactivated to undetectable levels within 5 minutes, 2.5 minutes, and 1 minute of heat treatment at 70, 80, and 90°C, respectively the suspension tests.	^26^	
Fumigant inactivation	VHP (10 ppm) TEG (2 ppm)	“Vapor concentrations of 10 ppm VHP or 2 ppm TEG can provide effective surface disinfection”	^29^	No
	Chlorine dioxide gas 0.03%	“Miura and Shibata (2010) described the efficacy of chlorine dioxide against the influenza virus. They suggested that chlorine dioxide has strong disinfectant activity at a concentration of 0.03%. Their review suggested that chlorine dioxide, either in a solution or gaseous form, could be effectively used to control influenza due to its strong antiviral effects used to control H1N1 infections”	^30,31^	
	Ethylene oxide gas	“H1N1 was completely inactivated by EO treatment in the surface tests.”	^26^	

AIVs, avian influenza virus; ELD, egg lethal dose; EO, ethylene oxide gas; HA, haemagglutinin; HID_50_, 50% human infectious dose; HK; HPAI, highly pathogenic avian influenza; LAIs, laboratory-acquired infections; LPAI, Low-pathogenic avian influenza; PMPS, potassium monopersulfate; PWs, poultry workers; QAC, quaternary ammonium compound; TCID_50_, 50% tissue culture infectious dose; TEG, triethylene glycol gas; VHP, hydrogen peroxide vapor; ZAI, Zoonotic avian influenza.

The efficacy of potassium monopersulfate (PMPS) in inactivating LPAI virus (strain A/duck/Aomori/Japan/395/2004 H7N1) was reported in the absence and presence of organic matter. PMPS at concentrations of 5000, 2500, and 1250 ppm^24^ reportedly could inactivate the virus even in organic materials within 5, 5, and 30 s, respectively. At 625 ppm, PMPS could inactivate the virus within 10 min without organic material; however, when organic matter was present, it could not inactivate the avian influenza virus within 15 min.^24^

A study by Hakim et al.^25^ reported successful disinfection and decontamination using a hypochlorous acid solution, where the aqueous phase of the original solution containing a free available chlorine concentration of 50 ppm could reduce the titer of ZAI virus (H7N1) from 10^7.7^ 50% tissue culture infectious doses per mL (TCID_50_/mL) to lower than the detectable limit within 5 s.^25^ ZAI H1N1 virus was completely inactivated in 1 min of 0.1 mol/L NaOH treatment in the suspension tests and effectively inactivated in the surface tests with the log reduction factor of 3.7.^26^

ZAI H5N1 virus was inactivated using soap (Lifebuoy^®^), detergent (Surf Excel^®^) and alkali (caustic soda) after 5 min at 0.1%, 0.2%, and 0.3% dilution.^27^ ZAI H1N1 virus was completely inactivated to undetectable levels within 1 min of treatment with 70% ethanol and 70% 1-propanol and 1 min of 0.1 mol/L NaOH.^26^ The commercial disinfectant Virkon^®^ at a 2% concentration is also reportedly effective for ZAI virus inactivation.^23,28^

Fumigation using vaporized hydrogen peroxide (10 ppm)^29^ and triethylene glycol (2 ppm),^29^ chlorine dioxide gas 0.03%,^30,31^ and ethylene oxide gas^26^ is effective for the inactivation of ZAI H1N1 viruses. Formaldehyde gas is a low-cost option in some countries for decontaminating large spaces and equipment. It is typically sublimated by diluting formaldehyde with water and heating it in a bucket on a hotplate instead of having more costly equipment with an automated program for maintaining chemical concentration over a set period.

Its use has been restricted in many countries as it is toxic and a listed carcinogen. Formaldehyde decontamination may leave a toxic residue that has to be neutralized and cleaned.^23^ Extreme care must be exercised to protect staff as the concentrations of many fumigants are immediately dangerous to life and health, and strict adherence to protocols is required to achieve the desired decontamination result. Decontaminating spaces using fumigants needs the spaces and equipment surfaces to be pre-cleaned and dried and a means to distribute the fumigant through the area to achieve uniform concentration and distribution and time of contact with surfaces while maintaining the required temperature and relative humidity.

##### Thermal and autoclaving

Thermal inactivation is generally effective for ZAI virus. De Benedictis et al.^23^ reported that heat treatment at 56°C to 60°C for 60 min would inactivate viral subtypes H5, H7, and H9. Thermal inactivation of HPAI A virus (H7N7) (>10^5^ PFU/mL) has been reported to be adequate at 63°C in 2 min.^32^ LPAIH1N1 virus was inactivated to undetectable levels within 5, 2.5, and 1-min heat treatment at 70, 80, and 90°C.^26^ In another study, the H5N1 virus reportedly lost infectivity after 30 min at 56°C and after 1 day at 28°C but remained viable for more than 100 days at 4°C.^27^ High-pressure treatments of HPAI virus (H7N7) (>10^5^ PFU/mL) were reported inactivating using 500 MPa at 15°C for 15 s.^32^

Evidence regarding the route of inoculation/modes of transmission, infectious dose, laboratory-acquired infections, and disinfection and decontamination strategies is provided in Table 1.

### Knowledge Gaps

#### Laboratory-acquired infections

No known laboratory-associated infections (LAIs) have been reported for ZAI virus. One of the challenges may be to discriminate between laboratory and community-acquired infections. In the United States, reporting laboratory-associated infections or exposure incidents for pathogens that are designated select agents is mandatory.^33^ HPAI strains (but not LPAI strains) are designated as Select Agents by the US Department of Agriculture (USDA) Veterinary Services (VS).^34,35^

If research with the ZAI virus involves recombinant nucleic acid work and the institution receives any funding from NIH, incident reporting to NIH is mandatory. Institutional and local authorities in some municipalities also mandate incident reporting for pathogens that are not select agents, but this requirement is inconsistent on a state and national level. Because public reporting of laboratory-associated infections or laboratory-based incidents that could result in ZAI virus infection is not universally mandatory, the opportunity to capture accurate and meaningful data to create an evidence base is limited.^33^

#### Infectious dose

Because the HID_50_ of human influenza viruses has been mainly determined using attenuated vaccine strains, it does not accurately represent what occurs in a natural infection. Direct HID studies in humans with wild-type strains would pose serious ethical challenges; however, additional animal studies with aerosol transmission could elucidate a more accurateHID_50_ in humans to better determine the actual workplace risk involved when working with ZAI virus.

#### Concentrations for chemical disinfections

In the case of chemical inactivation, the references provided in Table 1 describe various conditions, chemical agents, virus strains, and numerous variables. It would be beneficial to the biosafety practitioner to have a consensus view on the most appropriate concentrations and contact times and their application for common disinfectants. A caution about chemical incompatibilities that may compromise staff safety and in various diagnostic assays should be provided.

## Conclusions

Ideally, consensus guidance regarding contact time for commonly used chemical disinfectants at specific concentrations could be developed with particular attention to those available in low-resource settings. Having a more robust mandate to investigate suspect avian influenza LAIs and subsequently report cases would inform better risk management processes and prevent future exposures. Despite the lack of publicly reported ZAI virus LAIs, those working with HPAI ZAI viruses are required to use guidelines for Risk Group 3 respiratory pathogens to prevent exposure to and potential introduction into susceptible populations.

Reviewing all factors in an evidence-based risk assessment when conducting studies with LPAI and HPAI and addressing evidence gaps would provide appropriate biorisk management for laboratory-based tasks.

### Mycobacterium tuberculosis

#### General characteristics

*M. tuberculosis* is a bacterial agent belonging to the *Mycobacteriaceae* family. The disease caused by *M. tuberculosis* is known as Tuberculosis, commonly referred to as (TB). *M. tuberculosis* is a non-spore-forming bacillus^36^ classified as a risk group 3 pathogen.^37^ It is zoonotic,^36,38^ affecting humans, monkeys, parrots, cattle, sheep, goats, dogs, and cats^38^ and is endemic worldwide.^36,39^

#### Treatment and prophylaxis

TB infection is typically treated with combinations of antibiotics; isoniazid, ethambutol, rifampin, and pyrazinamide.^38,40,41^ Vaccination with Bacillus Calmette-Guérin, a strain *of Mycobacterium bovis*, is used as a prophylaxis in some countries where TB is common.^42^

#### Diagnosis

*M. tuberculosis-*related clinical and laboratory activities^43^ include the Mantoux tuberculin skin test or the TB blood test, sputum smear, and *in vitro* culture.^44^ Direct antimicrobial susceptibility testing (DST) using liquid cultures, especially the nitrate reductase assay, is considered the highest risk in the tuberculosis laboratory.^45^ Today, rapid tests such as GeneXpert have greatly reduced the requirement for in vitro culture to determine antibiotic sensitivity,^46,47^ significantly reducing the time to provide results. Such rapid tests have also considerably reduced the risks of laboratory-acquired infection associated with *M. tuberculosis in vitro* culture since sputum is mixed with a reagent and then added to a capsule inserted into a device that, within hours, determines infection and sensitivity to rifampin.

This is a tremendous improvement as *M. tuberculosis in vitro* culture determinations require 2–6 weeks incubation. In the United States, the GeneXpert test method is used, but cultures are also inoculated, since indeterminate results to rifampin resistance can occur.^48^ However, it should be highlighted that such simplified laboratory procedures only sometimes translate into feasibility to implement. Instead, the feasibility of GeneExpert testing depends on government commitment to ensure functioning infrastructure and stable power, supply of cartridges and functioning laboratory services, investment in expertise for handling (discordant) results, effective repair services, staff with monitoring capabilities, functioning sample transport, sustainable funding models and transparent donor agreements, and simple diagnostic algorithms.^49^

Consideration should also be given to the disposal method at the completion of the testing, and the manufacturer states that used cartridges should be disposed of according to institutions' standard practices.^50^ Recognizing that the GeneXpert cartridges contain infectious materials, they also contain chemicals that may not be compatible with autoclaving and alternative disposal methods should be used.

### Evidence

#### Route of transmission

Common modes of transmission are via inhalation of aerosols and droplets from handling infected specimens,^36^ percutaneous exposure (i.e., sharps, needlestick).^51,52^ Inhalation of aerosols/droplets occurs in clinical and domestic settings when patients cough; processing clinical samples containing *M. tuberculosis* may also generate aerosols and droplets. In addition, the formation of droplet nuclei carrying *M. tuberculosis* allows movement with air currents.

Aerosolization of this pathogen frequently occurs during autopsies, preparation of frozen sections of infected tissue, and procedures involving liquid cultures. The procedures conducted, the presence or absence of primary engineering controls, the concentration of *M. tuberculosis* in the specimen, and the ventilation in the laboratory are critical factors for the biorisk assessment. A study determined that the relative risk of TB infection of technicians doing acid-fast smear microscopy, compared with the general population, was 1.4, with a 95% confidence level (CI).

The same study found that the relative risk of TB infection in technicians doing Drug Susceptibility testing was 21.5. with a 95% CI.^53,54^ Infected animals can spread the infection to laboratory workers through aerosols, fomites, and bites and *vice versa.*^51,52^

#### Infectious dose

*M. tuberculosis* requires a very low infectious dose to initiate infection,^36^ with HID_50_ estimated at <10 bacilli.^36,55–57^

#### Laboratory-acquired infections

*M. tuberculosis* is in the top percentile of LAIs worldwide,^46^ with 194 cases and four deaths reported in the literature between 1930 and 1979. In addition, a literature survey published between 1979 and 2015 listed 255 laboratory-acquired infections and no fatalities.^58^

Most cases of laboratory-acquired tuberculosis arise from processing specimens obtained from infected humans. Naturally or experimentally infected nonhuman primates and other animals are potential tuberculosis infection sources for animal handlers and laboratory personnel.^57,59–61^ The risk of laboratorians becoming infected with tuberculosis is estimated at 8–30%.^62^ Aerosols present the most significant hazard, but infection can also occur from cutaneous injuries.^63^

In laboratory workers working with *M. tuberculosis*, the incidence of tuberculosis is three times higher than in those not working with the agent.^64,65^ The annual incidence of tuberculosis for laboratorians employed in Utah was 0.3 infections per 1000 people.^66^ The survey of British laboratory personnel by Grist, and Grist and Emslie from 1979 to 1989, reported an incidence ranging from 0.035 to 0.56 infections per 1000 people.^67–71^

Muller^63^ reported an incidence of 26.3 infections per 1000 people based on a survey of 77 TB laboratories in Germany, Austria, and Switzerland that was ∼100 times the frequency observed in the general population. Veterinary personnel involved in clinical and pathological examinations are also at a higher risk of infection.^72^ A review of *M*. *tuberculosis* infection, using tuberculin conversion in 17 Canadian acute care hospitals, indicated that the annual risk for laboratory workers was ∼1%, and the annual risk for pathology workers was 5.4%.

Laboratory exposures were associated with lower hourly air exchange rates (17 vs. 32.5 in labs without seroconversions). Exposures in pathology were associated with delayed diagnosis of patients; it was also noted that only half of the pathology work areas had more than 15 hourly exchange air ratesm.^73^

#### Disinfection/decontamination

##### Chemical

Numerous chemicals provide effective inactivation of *M. tuberculosis,* including phenol, povidone-iodine, chlorine, peroxide, aldehyde, and ethanol (70% ethanol)-based disinfectants (Table 2). Sodium hypochlorite at concentrations between 0.05% and 0.5% can be used for surface disinfection,^36^ provided the available chlorine concentration in the solution is 10,000 ppm (10,000 μg/mL) to ensure an adequate level of *M. tuberculosis* reduction.^74^ *M. tuberculosis* is also susceptible to chemical disinfectants such as N-dodecyl-1,3-propane diamine supplemented with sodium hydroxide, ethylene oxide, a mixture of 7.5% hydrogen peroxide, and 0.85% phosphoric acid, phenolics, 0.35% peracetic acid, orthophthaldehyde, or superoxidized water.^52^

**Table 2.** Detailed pathogen biosafety evidence for *Mycobacterium tuberculosis*

***Overview of the evidence and potential gaps in biosafety***				
***Method***	***Details***	***Evidence (direct quote where available)***	***Reference***	***Evidence gap? (yes/no)***
Route of inoculation	Inhalation (aerosols/droplets)	Transmission can be nosocomial or airborne (inhalation of droplet nuclei carrying *M. tuberculosis*, which are generated when patients with tuberculosis cough).	^36^	No
	Cutaneous transfer (fomites/bites/handling specimens)	Other modes of transmission include exposure to autopsy material, venereal transmission, and even percutaneous transmission (direct injury to the skin and mucous membranes through breaks in skin). Infected animals can spread the infection to laboratory workers through aerosols, fomites, bites.	^51,52^	No
		Most cases of laboratory-acquired tuberculosis arise from processing specimens obtained from infected humans. Naturally or experimentally infected nonhuman primates and other animals also are potential sources of tuberculosis for animal handlers and animal laboratory personnel.	^57,59–61^	
		Aerosols present the greatest hazard, but infection also can occur from cutaneous injuries.	^63^	
Infectious dose	<10 bacilli	The ID_50_ is estimated to be <10 bacilli in humans	^36,55–57^	No
LAIs	Top percentile of LAIs worldwide 200+ cases 4 deaths All reported infections: US—174 Britain—24 US and World—176	*Mycobacterium tuberculosis*, *Coxiella ÿurnetiid*, hantaviruses, arboviruses, hepatitis B virus, *Brucella* spp., *Salmonella* spp., *Shigella* spp., hepatitis C virus, and *Cryptosporidium* spp. Accounted for 1074 of the 1267 infections. 4079 LAIs were caused by 159 biological agents, although ten agents caused infections accounting for 50% of cases (brucellosis, Q fever, hepatitis, typhoid fever, tularemia, tuberculosis, dermatomycoses, Venezuelan equine encephalitis, psittacosis, and coccidioidomycosis). More than 200 cases of laboratory-acquired infections with *M. tuberculosis*, and *M. bovis* have been reported up to 1999. Up to 1976, 176 cases were reported with 4 deaths.	^57,104^	No
	0.3 infection per 1000 persons	The annual incidence of tuberculosis for laboratorians employed in Utah was 0.3 infection per 1,000 people.	^66^	
	0.035–0.56 infection per 1000 persons	The survey of British laboratory personnel by Grist and Grist and Emslie from 1979 to 1989 reported an incidence that varied from 0.035 to 0.56 infection per 1,000 people.	^67–71^	
	26.3 infections per 1000 persons	Muller reported an incidence of 26.3 infections per 1,000 people based on a survey of 77 tuberculosis laboratories in Germany, Austria, and Switzerland. This was approximately 100 times the frequency observed in the general population.	^63^	
		“An estimated 8%–30% of laboratorians may experience tuberculin conversions”	^62^	
	1939–1979 survey 1979–2015 literature survey	194 LAIs; 4 deaths. 255 LAIs; no deaths,	^58^	
	17 acute care hospital laboratories in Canada Support for “adequately ventilated spaces” recommended by WHO	“The risk of occupational tuberculosis (TB) infection and associated factors was estimated among all microbiology and pathology technicians and compared with a sample of nonclinical personnel in 17 Canadian acute care hospitals. Among participating lab workers, the average annual risk of tuberculin conversion was 1.0%. This was associated with lower hourly air exchange rates (16.7 versus 32.5 in workers with no conversion, *p* < 0.001) work in pathology (adjusted odds ratio [OR]: 5.4; [95% confidence interval: 1.3, 22].”	^73^	
	1970–1994 National Tuberculosis Program of Korea	“Compared to non-laboratory workers, the relative risk of TB was 1.4 (95%CI 0.2–10.0) among microscopy technicians and 7.8 (95%CI 1.7–34.9) among culture/DST technicians. TB developed among 7/15 DST technicians compared to only 2/59 culture/non-DST technicians. Compared to non-laboratory workers, the relative risk for DST technicians was 21.5 (95%CI 4.5–102.5).”	^53^	
Chemical inactivation	Amphyl and other phenol soap mixtures	Amphyl and other phenol soap mixtures and 0.05% to 0.5% sodium hypochlorite can be used for surface disinfection.	^36^	No
	Phenol (5% wt/vol)	“Phenol (5%) could reduce the titer of *M. tuberculosis* by 4 log1, in the suspension test as well as in the carrier test, even in the presence of sputum.”	^74^	
	Phenolic disinfectant 2 · 0 and 1 · 0% (v/v)	Phenol and phenol derivatives, known to be tuberculocidal even when organic matter is present. At concentrations of 2 · 0 and 1 · 0% (v/v), the disinfectants displayed the most rapid effects.	^75–77^	
	Sodium hypochlorite 0.05–0.5%	0.05% to 0.5% sodium hypochlorite can be used for surface disinfection.	^36^	
	Sodium hypochlorite 10,000 ppm	“Sodium hypochlorite required an available chlorine (Av Cl) concentration of 10,000 ppm (10,000 ug/ml) before an effective level of reduction could be obtained. Sodium hypochlorite required a minimum of 10,000 ppm of Av Cl to be effective against *M. tuberculosis*.”	^74^	
	Ethanol (70%)	“Ethanol (70%) proved to be effective against *M. tuberculosis* only in suspension in the absence of sputum.”	^74^	
		>3-log1o reduction in the titer of *M. tuberculosis* by 70% ethanol in a carrier test with a contact time of 15 min. Alcohols were the most effective agents against *M. tuberculosis*.	^76^	
	2.0% Glutaraldehydephenate (undiluted)	“Undiluted glutaraldehyde-phenate was clearly superior against *M. tuberculosis* and achieved an effective level of disinfection after 10 min of contact. Glutaraldehyde (2%) required a longer contact time to cause an effective reduction.”	^74^	
		“A 20-min exposure has been recommended as the minimum time needed to reliably kill *M. tuberculosis* with 2% alkaline glutaraldehyde.”	^80^	
	Povidone iodine (1.0% titratable 12)	“The povidone-iodine solution was highly effective against *M. tuberculosis* in the suspension test but was unable to inactivate it in the carrier test in the presence of sputum.”	^74^	
	2 or 4% Paraformaldehyde	“Incubation of *M. tuberculosis* in solutions containing 2 or 4% paraformaldehyde, the VesphineIIse solution, or 5% formalin killed all bacteria. These substances achieved 100% killing regardless of whether the *M. tuberculosis* was grown with or without Tween 80.”	^105^	
	3% Virkon^®^	“Therefore on the largely theoretical basis of test tube work 3% Virkon is required but in the real life world where both cleaning and disinfection take place 1% Virkon should be used for instruments contaminated with TB.”	^81^	
	Vaporized hydrogen peroxide for 90 min	“Initial inocula of *M. tuberculosis* (3 _log10_) and *Geobacillus stearothermophilus* (6 log_10_) were exposed to HPV at 10 locations during room experiments and both microorganisms were inactivated in all locations within 90 min of HPV exposure.” *M. tuberculosis* BIs were transferred to growth media at 15-min intervals during a 180-min HPV exposure period. No *M. tuberculosis* BIs grew following 30 min of HPV exposure.	^82^	
Thermal inactivation	80, 85, and 95°C for 20 min	“Using *Mycobacterium bovis* bacillus Calmette-Guérin (BCG) cultures and TB-positive sputum samples, we show that boiling for 20 min at 80, 85, and 95°C inactivates all *M. tuberculosis* bacilli.”	^106^	No, but definitive experiments demonstrating autoclave effectiveness are absent
	80°C for 20 min	“Our study has shown that heat inactivation performed at 80°C for 20 minutes using submerged suspensions of *M tuberculosis* in a water bath renders the samples safe for use by laboratory workers.”	^83^	
	100°C for 5 min	“This study showed that heating of cultures at 100°C for at least 5 min is sufficient to inactivate *M. tuberculosis*.”	^84^	
		“We have shown that heating of samples below 100 degrees C may not consistently kill mycobacteria; however, heating at 100 degrees C in a boiling-water bath or a forced-air oven for a minimum of 5 min kills mycobacteria, including *Mycobacterium thermoresistibile.*”	^107^	
Radiation inactivation	2450 MHz, 1.5 KW to heat the entire load to 100°C. Holding temperature and time for sterilizationwere set at 100°C for 30 min	“Before microwaving, samples containing acid fast bacilli (AFB) and live *M. tuberculosis* bacilli were 93.8% and 95% (≈94.7%) respectively; which came down to 14.2% (32) and <1% (≈0.9%) in post microwave.”	^89^	No
	UV at 254 nm at 40 erg per sec per mm	This investigation confirmed earlier reports that species of the genus *Mycobacterium* differ in their sensitivity to ultraviolet light irradiation (254 nm), The relative sensitivity to ultraviolet light de- creased in the following order: *M. tuberculosis (0040), M*. *[ortuitum* (0.25), *M. avium-intracellulare* (0.22), *M. phlei* (0.20), *M. marinum* (0.19), *M. kansasii* (0.18), *M. smegmatis* (0.16), and *M*. *flavescens* (0.11).	^87^	
	15 W General Electric G152 low-pressure mercury vapor germicidal lamp, yielding 34.29 μm/cm^2^ at 1 m. 810 μW s/cm^2^ (8100 ergs of energy)	To inactivate 90% of the *M. tuberculosis* and *M. marinum* cells, 7 and 22 sec of irradiation were required, respectively.	^86^	
	UV-C at 1104 μW s/cm^2^	“Sixteen tests were performed, with UV-C doses ranging from 276 to 1104 μW s/cm^2^. Mean (± SD) UV-C effectiveness ranged from 47.1% (±13.4) to 83.6% (±3.3). UV-C led to significantly greater inactivation of *Mycobacterium abscessus* (all *p*-values ≤0.045) than natural decay at all doses assessed”	^108^	No, but surrogate used

AFB, acid-fast bacilli; BCG, bacillus Calmette-Guérin; BI, biological indicator; DST, Direct antimicrobial susceptibility testing; SD, standard deviation; TB, tuberculosis; UV-C, ultraviolet-C (UV-C); WHO, World Health Organization.

One of the challenges for the disinfection of *M. tuberculosis* is the high level of organic matter when processing sputum samples. Phenol and phenol derivatives are tuberculocidal when organic matter is present at concentrations as high as 2.0 and 1.0% (v/v), displaying rapid bactericidal effects^75–77^ and 5% phenol effectively reduces the titer of *M. tuberculosis* by 4 log_10_ in the presence of sputum.^74^ Ethanol (70%) is effective against *M. tuberculosis* but only in the absence of sputum.

There was a greater than a 3log_10_ reduction in the titer of *M. tuberculosis* when treated with 70% ethanol for 15 min.^74^ A 1% povidone-iodine solution is highly effective against *M. tuberculosis* but could not inactivate it in the presence of sputum.^74^ Reports that alcoholic solutions of povidone-iodine have enhanced mycobactericidal activity and have been questioned.^78^

In both the absence and presence of sputum, undiluted glutaraldehyde-phenate used in a suspension test with a 1 min contact time and a carrier test with a 10-min contact time reduced the concentration of *M. tuberculosis* by 10^5^ and 10^4^ logs, respectively.^74,79^ A 20-min exposure is a minimum time to reliably kill *M. tuberculosis* with 2% alkaline glutaraldehyde.^79,80^ The commercial disinfectant 3% Virkon^®^ is also reportedly effective.^81^ Disinfection using vaporized hydrogen peroxide is effective following a 90-min dwell time.^82^

##### Thermal

Thermal means of inactivating *M. tuberculosis* are reportedly effective at 80°C for 20 min,^83,84^ 80, 85, and 95°C for 20 min or 100°C for 5 min.^84^ Validation of conditions and heat penetration for the specific sample type and volume is critical with thermal inactivation and explains the conflicting data published on temperature and time.^51^ Whether conducted with a flame or a slide warmer, the heat fixation procedure does not inactivate all *M. tuberculosis* bacilli in a smear, except for smears stained with the phenol-auramine method.^85^

##### Radiation

Physical disinfection of *M. tuberculosis* can be achieved using UV light, which can be used for surface disinfection.^36,52^ David et al.^86^ and David^87^ reported using UV at 254 nm to inactivate various *Mycobacterium* species, including *M. tuberculosis*. However, it is important to note that UV light bulbs require monitoring for intensity and frequent replacement when the germicidal wavelengths are no longer produced. The absence of shadows created by dust or fingerprints on the UV bulb is required for peak performance, and organic matter must be cleaned from work surfaces and equipment for effective decontamination.^88^

The application of microwave technology (2450 MHz, 1.5 KW to heat the load to 100°C for 30 min) has been successful in low-resource settings to minimize the bacterial burden in sputum samples containing acid-fast bacilli (AFB) and live *M. tuberculosis* bacilli.^89^

Evidence regarding the route of inoculation/modes of transmission, infectious dose, laboratory-acquired infections, and disinfection and decontamination strategies is provided in Table 2.

### Knowledge Gaps

#### Evidence for optimal autoclaving conditions

Effective use of the autoclave for waste decontamination depends on the type of autoclave (gravity displacement, positive pressure displacement, fuel-heated pressure cooker autoclaving, or pre-vacuum autoclave) and the density and loading of the waste. The WHO provides guidelines for using biological indicators to validate autoclaving conditions: “After a thorough risk assessment and validation, the following cycle will usually provide sterilization of correctly loaded autoclaves. Three minutes holding time at 134°C; 10 min holding time at 126°C; 15 min holding time at 121°C; 25 min holding time at 115°C.”^90,91^ A definitive study is required to appropriately validate these suggested conditions.

#### Evidence for *sputum* sample processing

Raw sputum or sputum sediments prepared with the NALC-NaOH or NaOH procedures recommended by the CDC are appropriate for use in GeneXpert assay.^50^ Other methods of liquefaction and chemical disinfection, such as 1:1 sputum and bleach,^92^ may not be compatible with further use of the sample in the GeneXpert assay; this should be researched with definitive guidelines written.

#### Evidence for the selection of engineering controls for diagnostic procedures

The requirement for primary containment is still debated for AFB smear procedures. Direct sputum-smear microscopy is a low-risk laboratory activity; however, it does have the potential to generate aerosols. The “low risk” determination is based on the viscosity of sputum samples, which reduces the risk of aerosolization and assumes that 90% of diagnostic acid-fast smears will be negative. However, a positive sputum sample may have 10^3^ to 10^8^ CFU.

Issues relating to the certification requirement for Class 1 BSC and the more complicated Class 2 BSC coupled with limited budgets in low-resource settings mean that there is a requirement for evidence-based risk assessment to determine the most appropriate engineering control for specific diagnostic or research laboratory procedures. The suggestion to use ventilated workstations to provide primary containment and directional ventilation without meeting a BSC standard has also been promoted.^93^

Key factors to consider for worker protection are (1) airflow into the device that draws aerosols away from the worker, with the inflow rate specified by the manufacturer, (2) the steps during a specific procedure that could result in aerosolization, and (3) HEPA filtration of exhaust air. A Class II biosafety cabinet is recommended for liquid cultures for direct antibiotic sensitivity testing (DST) or other procedures requiring HEPA-filtered air provided to the work surface.

The importance of verifying the airflow in a primary engineering control is illustrated in the case of three medical technicians who became infected in the same period. “The exposure was traced to a faulty microbiological safety hood. The hood was found to continuously circulate the contained and contaminated air rather than exhausting the air to the outside.” A case of endometrial tuberculosis and two other infections “could have been prevented by appropriate inspection and certification of the microbiologic safety hood.”^94^

#### Evidence for the selection of personal protection equipment

Good microbiological techniques in adequately ventilated areas, preferably inside a primary containment device that provides personnel protection, is a consensus recommendation. Good microbiological practice includes centrifugation using sealed rotors or safety covers. Personal protection equipment (PPE) does not replace primary engineering controls. The minimum clinical laboratory PPE would consist of a laboratory coat or gown, gloves, eye protection, and closed footwear.

In direct patient care, tuberculosis transmission is significantly reduced when staff wear respirators, and patients wear surgical masks. Studies on surgical masks versus N95 respiratory protection are needed to determine the appropriate protection during microbiology and pathology laboratory diagnostic procedures.

## Conclusions

The general biosafety guidelines for TB LAIs must be applied strictly: correct PPE, engineering controls, good microbiological practice, proper disposal, and an appropriate ventilation system to ensure a safe place of work for staff and the environment. While the need to perform TB diagnostics raises the risk profile of a laboratory, this can be managed by limiting or obviating the need for in vitro culture and focusing on AFB-staining and self-contained molecular diagnostic systems such as GeneXpert^95,96^ to provide a final diagnosis and antimicrobial resistance.
